# Children’s Nonverbal Displays of Winning and Losing: Effects of Social and Cultural Contexts on Smiles

**DOI:** 10.1007/s10919-016-0241-0

**Published:** 2016-09-16

**Authors:** Phoebe H. C. Mui, Martijn B. Goudbeek, Marc G. J. Swerts, Arpine Hovasapian

**Affiliations:** 1 Department of Communication and Information Sciences, Tilburg University, Postbus 90153, 5000 LE Tilburg, The Netherlands; 2 Department of Psychology and Social Behavior, University of California, Irvine, Irvine, CA 92697 USA

**Keywords:** Children, Context, Culture, Smile, Sociality

## Abstract

We examined the effects of social and cultural contexts on smiles displayed by children during gameplay. Eight-year-old Dutch and Chinese children either played a game alone or teamed up to play in pairs. Activation and intensity of facial muscles corresponding to Action Unit (AU) 6 and AU 12 were coded according to Facial Action Coding System. Co-occurrence of activation of AU 6 and AU 12, suggesting the presence of a Duchenne smile, was more frequent among children who teamed up than among children who played alone. Analyses of the intensity of smiles revealed an interaction between social and cultural contexts. Whereas smiles, both Duchenne and non-Duchenne, displayed by Chinese children who teamed up were more intense than those displayed by Chinese children who played alone, the effect of sociality on smile intensity was not observed for Dutch children. These findings suggest that the production of smiles by children in a competitive context is susceptible to both social and cultural factors.

## Introduction

Suppose a child played a game for a number of rounds, smiling upon winning and sulking upon losing. One might not find these reactions surprising, as they could be the kind of spontaneous nonverbal responses expected from a child under such circumstances. Now, suppose two children teamed up to play the same game, also winning in some rounds and losing in the others. Would they still smile upon winning and sulk upon losing? On the one hand, whether children played the game alone or not could have no influence on their facial expressions, for the rules and outcomes of the game remained the same. On the other hand, the fact that the game was played together with a teammate could augment the expressions displayed. The children were a team after all; their reactions to victory and defeat might have implications for the team spirit and rapport. Interestingly, the value placed on group harmony and cohesion is higher in some societies than in others, where self-expression and individual goals take precedence instead. Thus, whether children would smile or sulk might also depend on their cultural upbringing.

The susceptibility of children’s smiling behavior to social context and cultural context was addressed in the current study. Prior research has generated considerable knowledge about the effect of social context on smiles (e.g., Fridlund 1991; Kraut and Johnston 1979; Ruiz-Belda et al. 2003). However, the majority of such studies have been conducted on adults, not children. Cultural variability and universality in emotion expressions have also been investigated extensively (e.g., Elfenbein et al. 2007; Jack et al. 2012; Matsumoto et al. 2008); nevertheless, the effect of culture on smiling behavior itself has not received particular attention. In the few cross-cultural studies investigating the production of smiles (Marsh et al. 2007; Szarota 2010; Thibault et al. 2012), the smiles have been displayed by adults only, and the effect of social context has not been examined. The current study set out to investigate the interplay between social context and cultural context on smiles displayed by children upon winning and losing in games, thereby contributing to the existing knowledge about smiling behavior of children.

### Effects of Sociality and Culture on Adult’s Smiling Behavior

People may express their affective state as it is, conceal it, or amplify it (Ekman et al. 1972; Ekman and Oster 1979), contingent upon various factors. One such factor is the immediate social context people find themselves in. For example, happy soccer fans and pedestrians enjoying a sunny day smiled more when they were engaged in social interaction than when they were not (Kraut and Johnston 1979; Ruiz-Belda et al. 2003). Amongst people who viewed a pleasant videotape either alone or with a friend, occurrences of smiles were also better predicted by sociality than by the actual happiness experienced (Fridlund 1991). Being engaged in social interactions influences not only expressions of affective states in general, but also smiles, which are characteristic expressions associated with accomplishment in competitive contexts (Matsumoto and Willingham 2006). Amateur bowlers who made a good roll smiled more whilst facing their friends than whilst facing the pins (Kraut and Johnston 1979; Ruiz-Belda et al. 2003). Professional judo athletes who were awarded a medal in the Olympics also produced most smiles—specifically, Duchenne smiles (Duchenne 1862/1990)–during the interactive stage of the awards ceremony (Fernández-Dols and Ruiz-Belda 1995), a finding recently replicated by Crivelli et al. (2015). Duchenne smiles involve both the muscles lifting the corners of the mouth and the muscles orbiting the eyes. They have been widely believed to be genuine smiles that reflect positive affect; however, recent literature has shown that people are able to produce Duchenne smiles deliberately (e.g., Gosselin et al. 2010; Gunnery et al. 2013; Krumhuber et al. 2014; Krumhuber and Manstead 2009). Non-Duchenne smiles, which involve only the muscles lifting mouth corners, have been regarded as masking smiles (Ekman and Friesen 1982) often displayed in the absence of positive affect.

Are smiles also displayed upon losing, and if so, are they susceptible to the effect of sociality as well? In their study on Olympic judo medalists, Matsumoto and Willingham (2006) argued that whereas gold and bronze medalists could be regarded as winners, silver medalists could be considered losers, for it was possible that they experienced regret over having just missed out on the gold medal (Medvec et al. 1995). In line with this assertion, the type of smiles displayed by the medalists indeed varied as a function of both sociality and the medal received. Overall, medalists smiled more during the interactive stages of the awards ceremony than during the non-interactive stages. However, whereas gold and bronze medalists displayed Duchenne smiles, silver medalists were more likely to display non-Duchenne smiles. During the non-interactive stage, some silver medalists even displayed negative emotions or expressions that were uninterpretable.

Other than social context, cultural context has been shown to influence affective behavior of adults as well. In the unpublished but widely cited dissertation by Friesen (1972, as cited in e.g., Fischer and Manstead 2000; Matsumoto and Ekman 1989; Niedenthal et al. 2013), American and Japanese participants watched distressing films alone or in the presence of the experimenter. No cultural difference was found for participants watching the films alone; yet, in the presence of an experimenter, only Japanese masked their negative emotions. This finding could be attributed to the notion of display rules, which are social norms governing the management and modification of expressive behavior depending on social circumstances (Ekman and Friesen 1969). Display rules vary across cultures, as cultures differ in the values and norms they prescribe. For example, display rules have been found to relate to the cultural dimension of individualism-collectivism (Hofstede 2001). Members of individualistic cultures (such as United States and the Netherlands) tend to define themselves as unique individuals whose personal goals precede those of the collective, whereas members of collectivistic cultures (such as Japan and China) define themselves as part of the collective and tend to prioritize group concerns over personal ones (Markus and Kitayama 1991; Triandis 1995; see Oyserman et al. 2002, for a meta-analysis). In accordance with this cultural framework, individualism has been found to correlate positively with higher expressivity norms, suggesting that members of individualistic cultures prefer displaying affective state as it is over concealing it; members of collectivistic cultures tend to conceal negative affective states that might hinder group harmony (Matsumoto et al. 2008; Rychlowska et al. 2015). Another factor that contributes to cultural differences in display rules is the notion of tightness-looseness (Pelto 1968). In countries with a tight culture (such as China and Singapore), solid social norms exist; behavior that deviates from the norms is reprehended. On the contrary, in countries with a loose culture (such as the Netherlands and New Zealand), norms are more ambiguous and the level of tolerance for norm violation is higher. Because of the lower degree of situational constraint in loose societies, expressive displays of affect in public (e.g., laughing out loud on a bus) are considered more appropriate by members of loose societies than by members of tight societies (Gelfand et al. 2011).

As discussed above, prior research has shown support for the effect of cultural context on emotion expression in general. By contrast, less evidence has been garnered for the effect of culture on smiling behavior specifically. Szarota (2010) examined smiles displayed by Europeans and found that members of former Soviet countries smiled less than members of Western European countries. However, the smiles in question were obtained from profile pictures of social media users; they did not stem from competitive contexts. Most of the studies considering smiles as expressions of winning and losing (i.e., smiles of Olympic medalists as described earlier) were based on a monocultural sample. The sample in the study by Matsumoto and Willingham (2006) was culturally diverse but as the sample size per country was too small, countries were classified into three groups (East Asia, North America-Western Europe, or others) for analyses. During the non-interactive stage of the awards ceremony, gold and bronze medalists from East Asian and North America-Western Europe were more likely to display Duchenne smiles, whereas those from other countries were more likely to display non-Duchenne smiles. Such grouping of countries was arguably arbitrary and might have disregarded nuanced differences among countries that are geographically close. It remains unclear how cultural values manifest themselves in smiles displayed in competitive situations.

### Effects of Sociality and Culture on Children’s Expressions

The effect of sociality on nonverbal expressions can be observed among not only adults but also children. Upon listening to humorous stimuli, seven-year-old children smiled significantly more when another smiling child was present, compared to when they were alone (Chapman 1973). Both younger and older preschoolers (average age of 59 and 75 months, respectively) displayed more smiles when unwrapping an undesirable gift in the presence of the experimenter, compared to those who unwrapped the gift alone (Josephs 1994). Indeed, conformity to display rules, as prescribed by social and cultural norms, is crucial for successful social development and is evident in children as young as three (Denham et al. 2015). The mastery of display rules develops with age (e.g., Gnepp and Hess 1986; Halberstadt et al. 2001; Lawton et al. 1992; Saarni 1999; Zeman and Garber 1996). For example, Saarni (1984) found that when unwrapping a disappointing gift alone, third- and fifth-graders smiled more than first-graders as a means of regulating negative emotion.

Cultural variability in the use of display rules by children has been reported as well (Cole et al. 2002; Garrett-Peters and Fox 2007; for a review, see Halberstadt and Lozada 2011). Nevertheless, prior research has shed little light on the extent to which smiles displayed by children in competitive contexts are subject to sociality as well as culture. Schneider and Josephs (1991) observed an effect of sociality on expressions of German preschool children playing a competitive game: Children smiled more when they were playing a game with the experimenter than when they were playing alone; occurrences of smiles were better predicted by sociality than by outcome of the game. However, cultural comparison could not be made as it was a monocultural study; no distinction between Duchenne smiles and non-Duchenne smiles was made either. Shahid et al. (2008) elicited expressions from Dutch and Pakistani children who played a game either alone or in pairs, which were used as stimuli in a subsequent perception task. In the perception task, adult judges only observed one child at a time and were asked to indicate whether the child had won or lost. Judges performed better when judging Pakistani children who were playing in pairs than Pakistani children playing alone; whether or not Dutch children were playing in pairs or alone did not influence how they were judged. Although these findings hinted at cultural differences in facial expressions displayed in a competitive context, the expressions were not coded by Shahid et al. (2008); thus, it could not be ascertained whether cultural differences existed at the level of spontaneous expression production, and whether or not culture influenced the production of smiles.

### Present Study

In the present study, we set out to examine the possible interplay between social and cultural factors on smiling behavior of children, displayed in a competitive context. We were interested in whether or not the effects of sociality and culture on smiles displayed by adults, as reported in prior research, would be evident among children as well. In view of the relatively limited amount of prior research devoted to smiling behavior exhibited in competitive situations, we aimed to investigate if and how children would moderate their displays of victory and defeat, as a function of social and cultural context.

To this end, we employed the game paradigm used by Shahid et al. (2008) to elicit expressions associated with winning and losing during gameplay. Specifically, we recruited eight-year-olds from the Netherlands and mainland China to play a competitive game either alone against a computer, or teamed up with a friend who was physically present, also against a computer. We chose to employ this game paradigm as it had been shown to be a naturalistic way of engaging children from distinct cultural groups, as evident in the spontaneous facial expressions elicited. Moreover, the paradigm was based on a simple number guessing game which did not involve any culture-specific knowledge or procedure. As a result, method bias (e.g., Van de Vijver and Leung 1997, 2001) was prevented, warranting equivalence and validity of the present cross-cultural study. We elected to compare Dutch and Chinese as these two cultural groups differ markedly on dimensions underlying cultural differences in display rules that govern which affective expressions are appropriate in varying situations: individualism-collectivism (out of 70 countries, rank of the Netherlands: 4, rank of China: 53; Hofstede 2001) and tightness-looseness (out of 33 societies, rank of the Netherlands: 29, rank of China: 9; Gelfand et al. 2011). Individualism-collectivism is also one of the cultural frameworks identified by Halberstadt and Lozada (2011) as a contributing factor to the socialization of emotion development in children. The findings by Shahid et al. hinted at differences in emotion expression by Dutch children and Pakistani children; we wondered if such differences would also be evident between Dutch and Chinese, as both Chinese and Pakistani cultures are relatively more collectivistic (rank of Pakistan: 64; Hofstede 2001) and tighter than the Dutch culture (rank of Pakistan: 1; Gelfand et al. 2011). We decided to study the expressions of eight-year-olds as it was evident from previous studies that children at this age already demonstrate awareness and use of display rules, as described in the previous section.

The elicited smiling behavior of children was coded in accordance with the Facial Action Coding System (FACS; Ekman and Friesen 1978). The action units (AU) activated in smiling behavior are AU 6 and AU 12, which activate the orbicularis oculi muscle and the zygomatic major muscle, respectively. The occurrence and intensity of these action units were coded by a certified FACS coder, as per the updated FACS manual guidelines (Ekman et al. 2002).

Based on previous research on the effect of sociality on smiling, we expected that overall, children who paired up would display more smiles than children who played the game alone. Moreover, we explored potential differences in smiles displayed by Dutch and Chinese children, on the basis of the importance of the collective and group harmony in China and the cultural emphasis on the independent unique self in the Netherlands (e.g., Gelfand et al. 2011; Hofstede 2001; Markus and Kitayama 1991; Matsumoto et al. 2008). Shahid et al. (2008) found that people were better at recognizing the game outcome from expressions of Pakistani children who paired up than from expressions of Pakistani children playing alone; no such advantage was observed in recognizing the game outcome of Dutch children. As the Chinese and Pakistani cultures are similar in their emphasis on group harmony, we wondered if culture would interact with sociality in our study too. Specifically, we investigated if difference between smile intensity observed in the social and non-social condition, if any, would be smaller among Dutch children.

## Method

### Participants

Fifty-five Chinese (34 male) and 31 Dutch (23 male) participants took part in our study, with the informed consent of their parents and school teachers. The Chinese participants were 8-year-old pupils at a school in Suzhou, China. The Dutch participants were 8-year-olds attending a school in Tilburg, the Netherlands.

### Task and Procedure

We used a game paradigm (Shahid, et al. 2008) to elicit facial expressions associated with winning and losing. Children were randomly assigned to one of the two conditions: playing the game alone (15 Dutch, 13 Chinese), or pairing up with a friend of their choice who was physically present (16 Dutch pairs, 21 Chinese pairs). All children assigned to play in pairs chose to pair up with a friend of the same gender, with whom they had to collaborate throughout the game. Children were seated in front of a computer screen and videotaped by a camcorder placed on top of the screen. Those assigned to pair up sat next to their game partner, and could interact with each other freely. For subsequent coding and analyses, only one child from each pair was randomly selected; the other child was not visible in the recordings. Before the start of the game, an experimenter gave instructions to children in their native language (Dutch or Chinese) to ensure that they understood the rules of the game.

**Fig. 1:**
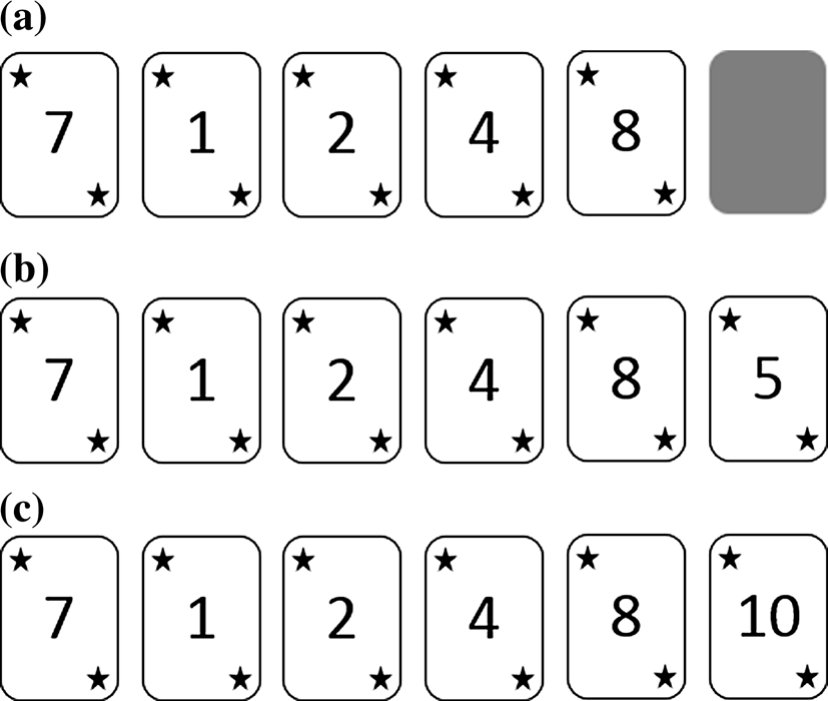
An example of the set-up of a game round. The task of the children was to **a** indicate whether the next hidden number would be higher or lower than the last number shown. The last card in every game round was manipulated to induce **b** winning, as the number on the last card is likely, given the preceding number; and **c** losing, as the number on the last card is unlikely, given the preceding number

The game was a simple number guessing game consisting of six rounds. In each round, six cards were displayed on a computer screen but initially only the number on the first card was shown. The task of children was to indicate whether the number on the next card, which could range from one to ten, would be higher or lower than the number currently shown. An example set-up of a game round is shown in Fig. 1a. Children who played in pairs were asked to collaborate with each other to decide on an answer. The rules were as follows: To win a round, children needed to give correct answers for all six cards within that round. When they gave an incorrect answer for any card before the sixth card, that round ended immediately and was considered an incomplete, losing round. When they gave an incorrect answer only for the sixth card, that round was considered a completed, losing round. For reasons of comparability, incomplete losing rounds were not included into subsequent analyses; as explained below, only completed rounds, both winning and losing, were considered.

Unbeknownst to the children, we manipulated the number on the last card in each round, ensuring that both wins and losses were inevitable. In the winning variant (Fig. 1b), the number on the last card was a likely number given the preceding number (e.g., five preceded by eight), based on consideration of the possible range of numbers (one to ten). On the contrary, in the losing variant (Fig. 1c), the number on the last card was unlikely given the preceding number (e.g., ten preceded by eight). This manipulation resulted in at least two wins and two losses for all children.

### Video Recordings

The elicitation task described above gave rise to a considerable number of video recordings, which showed a frontal view of the children roughly from their shoulders upward. For each child or pair, we only retained recordings obtained from two rounds: the second win and the second loss. This was done to standardize the recordings across children, as well as to rule out unfamiliarity and boredom with the game as a confounding factor for the expressions displayed by children. Such a selection resulted in two recordings per participant or pair; 56 recordings from participants who played alone (26 Chinese, 30 Dutch) and 74 from pairs (42 Chinese pairs, 32 Dutch pairs) were retained. We trimmed the recordings in length such that the onset showed the moment at which children had just given their answer for the last card. The recordings ended with the moment at which children’s response to seeing the game outcome had subsided. On average, a video fragment lasted five seconds.

### FACS Coding

The facial expressions elicited were analyzed using FACS (Ekman and Friesen 1978), which identifies the presence and intensity of facial muscle movements, termed action units (AU). AU 6 and AU 12, which are activated in smiling behavior, were coded in the present study. They were coded on a 6-point scale with 0 indicating absence of activation and higher scores indicating more intense activation.

A certified FACS coder who was blind to the hypotheses and the conditions of the experiment viewed and analyzed all expressions. Video recordings were muted to avoid the possibility that children’s talk could influence coding. Coding criteria was based on the updated FACS manual (Ekman et al. 2002), which has more stringent criteria on coding AU 6 in the presence of AU 12. Before coding, the exact time of onset of facial expression was identified. In four cases, the child presented more than one facial expression during the recording. For these cases, the first expression was used for analyses, as it showed the initial spontaneous response. In order to test reliability, a second coder analyzed a subset (25 %) of the recordings. Thirty-five expressions were coded by both coders. The intraclass correlation coefficient (ICC) indicated acceptable inter-rater reliability for AU 6 (ICC = .83) and AU 12 (ICC = .86). Of the 130 recordings coded, four (3.08 %) were assigned a missing value, as the facial expression could not be determined due to video quality.

FACS codes were recoded into new variables, indicating whether a smile was present or not, and whether the smile coded was Duchenne or non-Duchenne. A Duchenne smile was defined as the co-activation of AU 6 and AU 12, whereas a non-Duchenne smile was defined as the activation of AU 12 only, in the absence of AU 6.

## Results

The FACS codes were analyzed as follows: First, we investigated the conditions (social context: alone or in pairs; cultural context: Dutch or Chinese; game outcome: win or loss) in which smiles were more likely to be present than absent. Second, among cases where smiles were present, we compared the occurrences of Duchenne smiles and non-Duchenne smiles in different conditions. Last of all, we analyzed the level of intensity of overall smiles, as well as for AU 6 and AU 12, respectively.

### Distribution of Smiles

**Fig. 2:**
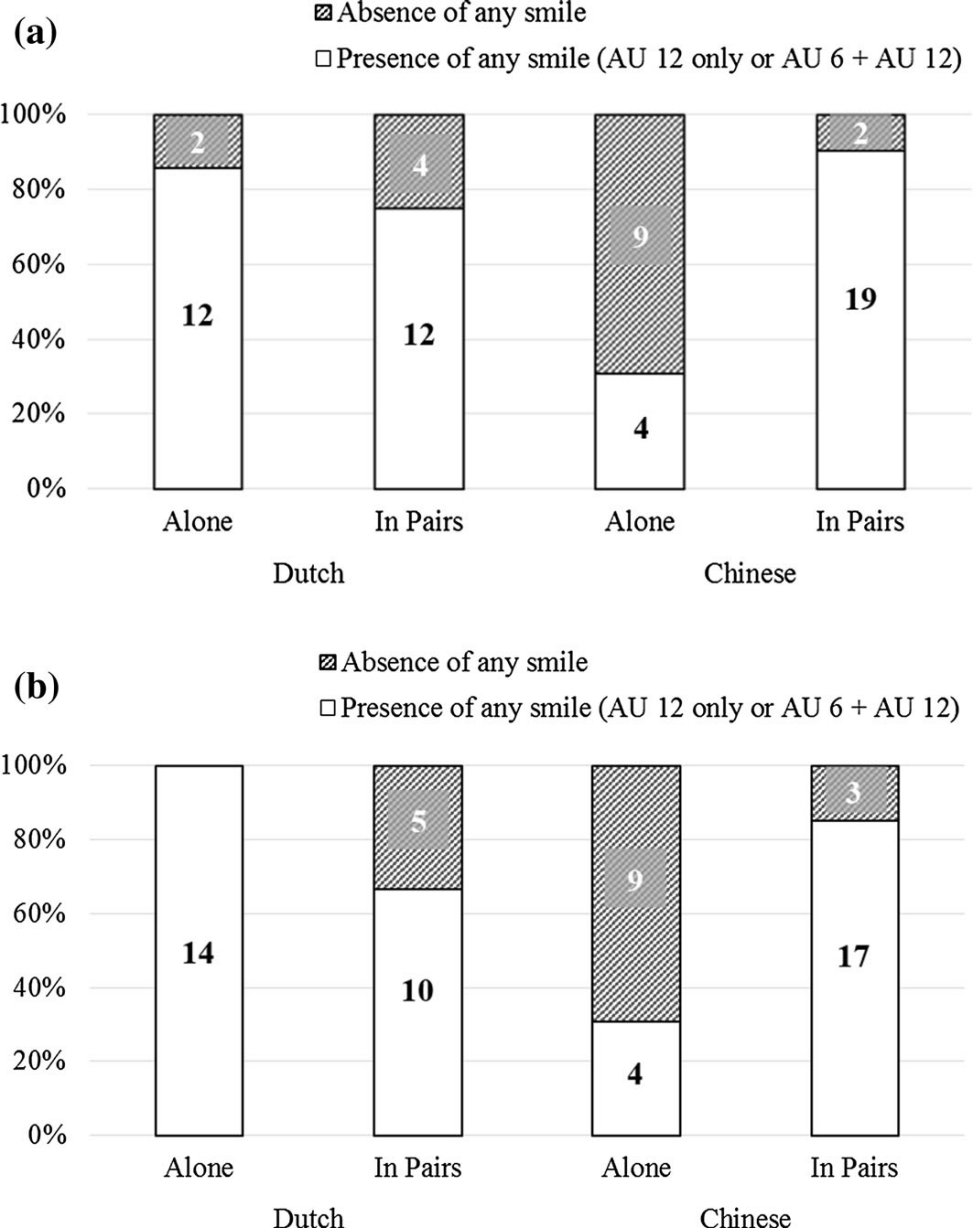
Distribution of smiles and non-smiles as a function of sociality and culture, for video recordings depicting **a** winning, and **b** losing, respectively

A smile was defined as the activation of AU 12, without regard for AU 6. Amongst the 126 codeable recordings elicited in this study, a smile was observed in 92 cases (73.02 %). The distribution of the presence and absence of smiles across conditions is illustrated in Fig. 2. In addition, the distribution of smiles across conditions is listed in Table 1; cases in which no smile was observed were omitted, so that the proportions were calculated based on cases in which a smile was observed (*N* = 92) rather than the overall sample.

**Table 1:** Frequency and percentages of smiles observed as a function of sociality, culture, and game outcome

	Dutch	Chinese	Total
	Win		
Alone	12 (13.04 %)	4 (4.35 %)	16 (17.39 %)
Pairs	12 (13.04 %)	19 (20.65 %)	31 (33.69 %)
Total	24 (26.08 %)	23 (25.00 %)	47 (51.08 %)
	Loss		
Alone	14 (15.22 %)	4 (4.35 %)	18 (19.57 %)
Pairs	10 (10.87 %)	17 (18.48 %)	27 (29.35)
Total	24 (26.09 %)	21 (22.83 %)	45 (48.92 %)

Percentages are calculated based on the total number of smiles (*N* = 92), observed across all conditions

Chi-square tests were conducted to examine the relation between presence of smiles and each of the factors examined in this study, namely social context (alone or in pairs), cultural context (Dutch or Chinese), and game outcome (win or loss). Presence of smile was significantly associated with the social context in which children played the game, Pearson’s χ^2^ (1) = 4.85, *p* = .028, φ = .20. Children were more likely to smile when they paired up than when they played alone (odds ratio = 2.44). Presence of smile was also associated with cultural context, the significance of which was marginal, Pearson’s χ^2^ (1) = 3.92, *p* = .048, φ = .18. The odds of Dutch children smiling were 2.28 times higher than those of Chinese children.

Whereas Pearson’s chi-square tests were appropriate for the between-subject factors (social context and cultural context), a McNemar’s chi-square test (McNemar 1947) with Yates correction was conducted for the within-subject factor (game outcome) to account for the dependence in data. Results from the McNemar’s test showed that game outcome was not significantly associated with the presence of smile, McNemar’s χ^2^ (1) = 0.063, *p* = .80, φ = .032. Thus, whether children smiled or not was not related to whether they were winning or losing.

**Fig. 3:**
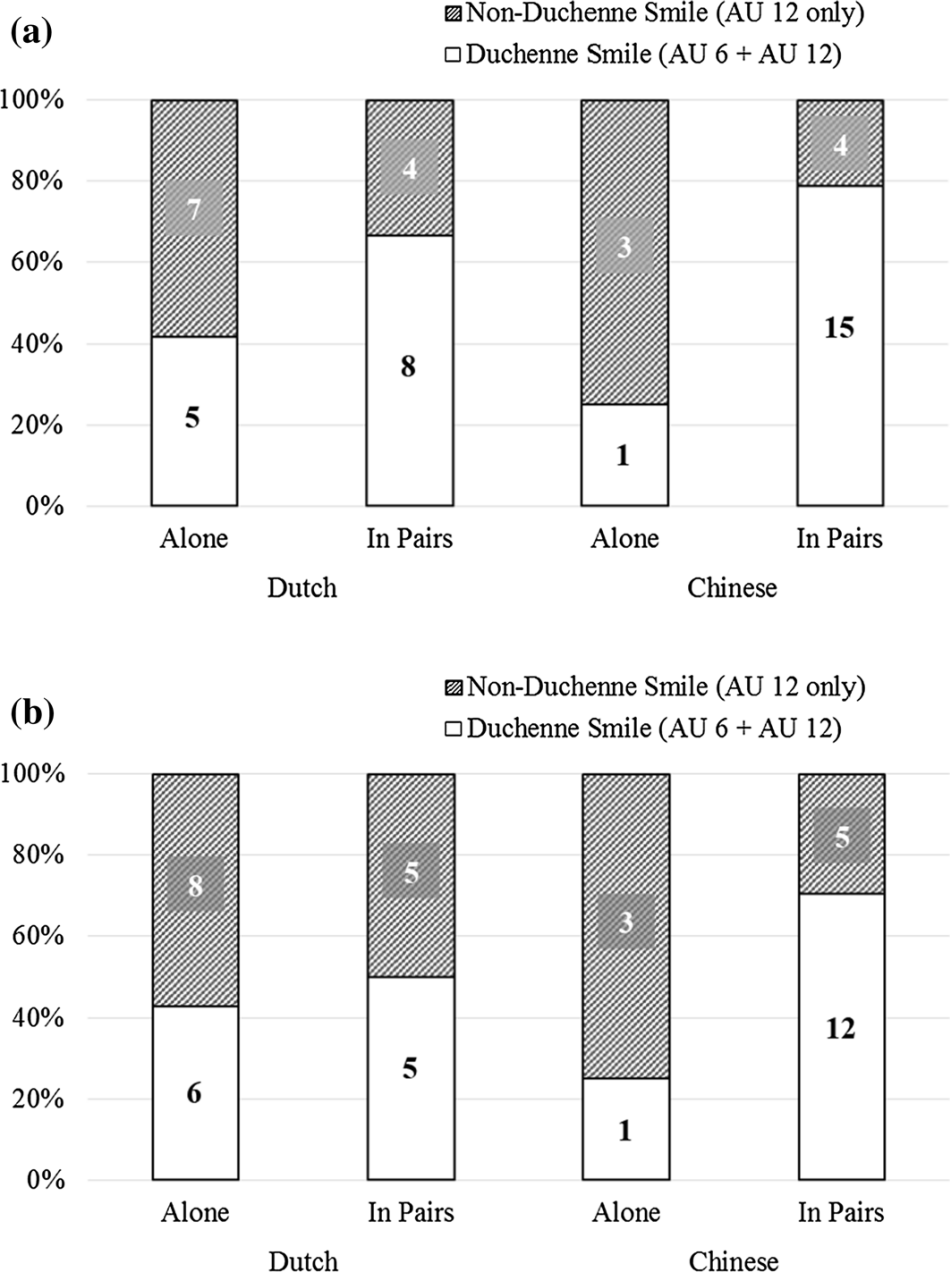
Distribution of Duchenne smiles and non-Duchenne smiles as a function of sociality and culture, for video recordings depicting **a** winning, and **b** losing, respectively

As reported above, a smile was observed in 73.02 % of the codeable recordings. We refined our analyses by distinguishing Duchenne smiles from non-Duchenne smiles. A smile was coded as Duchenne when both AU 6 and AU 12 were activated, and as non-Duchenne when only AU 12 was activated. The distribution of Duchenne smiles and non-Duchenne smiles across conditions are illustrated in Fig. 3.

Overall, Duchenne smiles accounted for 57.61 % of the smiles observed. Smile type was significantly associated with the social context in which children played the game, Pearson’s χ^2^ (1) = 8.29, *p* = .004, φ = .30. Children were more likely to display a Duchenne smile when they played in pairs than when they played alone (odds ratio = 3.59). Cultural context was not significantly associated with smile type, Pearson’s χ^2^ (1) = 2.38, *p* = .12, φ = .16, suggesting that Dutch and Chinese were equally likely to display either type of smile. Likewise, game outcome was not significantly associated with the type of smile, McNemar’s χ^2^ (1) = 1.23, *p* = .27, φ = .18. Thus, whether children won or lost was not related to the type of smile displayed either.

### Intensity of Overall Smile, AU 6, and AU 12

Intensity of activation in AU 6 and AU 12 was coded on a 6-point scale, with higher scores indicating more intense activation. A measure of overall smile intensity was computed by adding up the level of activation of AU 6 and that of AU 12 (for previous works adopting the same procedure, see Harker and Keltner 2001; Josephs 1994; Wojcik et al. 2015). Means and standard deviations of the levels of intensity by condition are listed in Table 2.

**Table 2:** Mean overall smile intensity and standard deviations as a function of sociality, culture, and game outcome

	Dutch		Chinese	
	*M*	*SD*	*M*	*SD*
	Win			
Alone	2.21	2.12	0.62	2.11
In pairs	2.73	2.12	4.65	2.12
	Loss			
Alone	2.71	2.29	0.62	2.29
In pairs	2.20	2.29	3.40	2.29

**Fig. 4:**
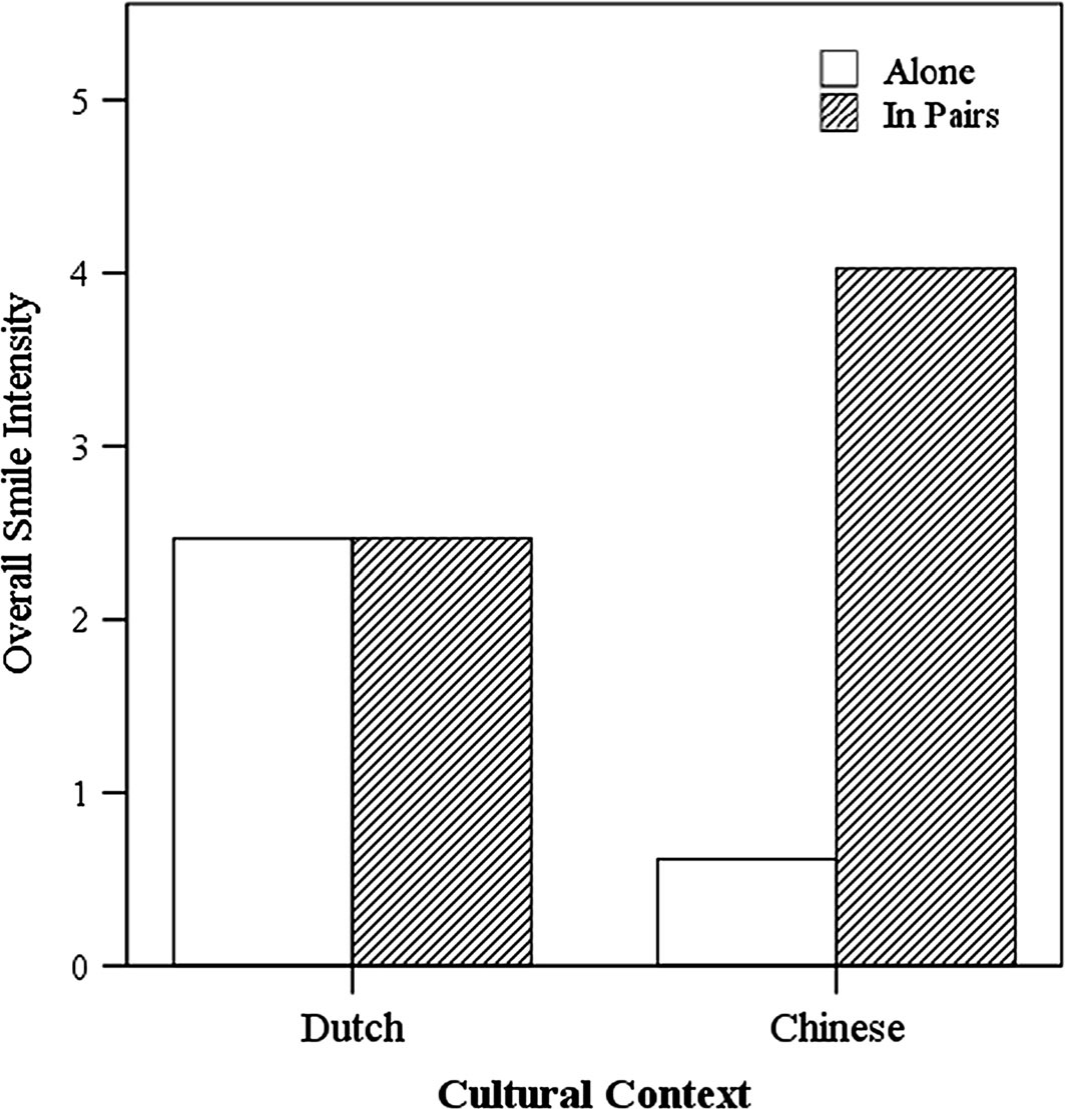
Overall smile intensity as a function of social context (alone or in pairs) and cultural context (Dutch or Chinese)

To account for the mixed factorial design of our study, 2 (social context: alone or in pairs) × 2 (cultural context: Dutch or Chinese) × 2 (game context: win or loss) repeated-measures ANOVA were conducted for overall smile intensity, AU 6, and AU 12. A significant main effect of social context on overall smile intensity was observed, *F*(1,58) = 13.79, *p* < .001, ŋ_*p*_^2^ = .19. This effect was qualified by an interaction with cultural context, *F*(1, 58) = 13.75, *p* < .001, ŋ_*p*_^2^ = .19, as shown in Fig. 4. Smiles displayed by children playing in pairs (*M* = 3.25, *SD* = 1.80) were more intense than those displayed by children playing alone (*M* = 1.54, *SD* = 1.79). However, as shown in the interaction, this finding was obtained for Chinese only but not for Dutch, *M* difference = 3.41, *SD* = 1.24, *p* < .001. The smiles displayed by Dutch children who played in pairs were equally intense as those displayed by Dutch children who played alone, *M* difference = 0.002, *SD* = 1.03, *p* = 1.00. No other significant effect emerged from the ANOVA.

The same pattern of results were observed for AU 6. A repeated-measures ANOVA revealed a significant main effect of social context on the activation intensity of AU 6, *F*(1, 60) = 11.69, *p* = .001, ŋ_*p*_^2^ = .16, which was qualified by an interaction with cultural context, *F*(1, 60) = 7.21, *p* = .009, ŋ_*p*_^2^ = .11. Higher activation intensity of AU 6 was coded for cases where children were playing in pairs (*M* = 1.20, *SD* = 0.96) than cases where children were playing alone (*M* = 0.37, *SD* = 0.96). However, this effect was only observed for Chinese children, *M* difference = 1.47, *SD* = 1.24, *p* < .001. Activation intensity of AU 6 did not differ between Dutch children who played in pairs and Dutch children who played alone, *M* difference = 0.18, *SD* = 1.03, *p* = .61.

Findings observed for AU 12 were also consistent with the results reported above. As was the case with overall smile and AU 6, higher intensity of AU 12 was coded for cases where children were playing in pairs (*M* = 2.09, *SD* = 0.98) than cases where children were playing alone (*M* = 1.14, *SD* = 0.97), *F*(1, 59) = 14.71, *p* < .001, ŋ_*p*_^2^ = .20. Again, social context interacted with cultural context, *F*(1, 59) = 15.23, *p* < .001, ŋ_*p*_^2^ = .21, the effect of which was present for Chinese children only, *M* difference = 1.91, *SD* = 1.27, *p* < .001. Regardless of whether Dutch children played in pairs or alone, the activation intensity of AU 12 was the same, *M* difference = 0.02, *SD* = 1.04, *p* = .96.

## Discussion

We examined the frequency and intensity of smiles displayed by Dutch and Chinese children in a game played either alone or in pairs. Based on the literature on effects of sociality on smiling behavior (e.g., Fridlund 1991; Kraut and Johnston 1979; Ruiz-Belda et al. 2003), we expected more smiles from children who paired up than children who were alone. Indeed, the frequency of smiles was higher for children who paired up than that of children playing alone. In addition, we compared the smiles displayed by Dutch and Chinese, two cultural groups which are distinctly different in terms of individualism-collectivism and tightness-looseness (e.g., Gelfand et al. 2011; Halberstadt and Lozada 2011; Hofstede 2001; Markus and Kitayama 1991; Matsumoto et al. 2008). Specifically, we wondered if the effect of sociality would be more prominent among Chinese children than among Dutch children. Indeed, the intensity of smiles displayed by children varied as a function of sociality and culture. Higher intensity was observed for smiles displayed by Chinese children who paired up than by Chinese children playing alone; such an effect of sociality was not observed for Dutch children. These results bear some resemblance to the findings of a previous study employing the same paradigm (Shahid et al. 2008), in which expressions were elicited from Dutch and Pakistani children who played the game in pairs or alone. The expressions served as stimuli in a subsequent perception test, where adult judges were asked to recognize the game outcome experienced by the children on the basis of the elicited expressions. Judges performed better when judging Pakistani pairs than Pakistani individuals; whether or not Dutch children were playing in pairs or alone did not influence the judgment of Dutch children. In a similar vein, we have observed that the effect of sociality was more prominent among the cultural group that is relatively tighter and more collectivistic (Chinese) than the cultural group that is looser and more individualistic (Dutch).

Interestingly, as revealed by the chi-square analysis, the outcome of the game was not related to whether or not children smiled. Children were equally likely to smile and not to smile, irrespective of whether they had just won or lost. These findings might appear surprising, as one might expect children to smile more upon winning than upon losing, other things being equal. A possible explanation concerns how we manipulated the losing instances. As described earlier, in half of the game rounds, we manipulated the number on the sixth card such that it was an unlikely number given the preceding number (e.g., ten preceded by eight). Giving an incorrect answer resulted in a loss for every child; yet, children might have differed in how they subjectively perceived this loss. Some children might have been upset by this defeat and did not smile. Others might have been confused by the loss and became uncertain of themselves; as a result, they might have smiled, for smiles have been found to be a cue of uncertainty among adults (Swerts and Krahmer 2005) as well as children (Visser et al. 2014). We did attempt to circumvent such confounds by running practice trials with the children, as well as by analyzing expressions displayed during the second win and the second loss only, thereby ensuring that unfamiliarity with or surprise about the game would not influence subsequent findings. To obtain a better understanding of how children perceive the wins and losses, researchers interested in employing this game paradigm could consider assessing the subjective experience of children about the winning and losing rounds.

Our study contributes to the understanding of smiling behavior of children in a number of ways. First, to the best of our understanding, it is a pioneering study devoted to investigating how the production of smiles by children in a competitive context is susceptible to both social and cultural factors. Prior research focused on a subset of the factors examined in our study, such as smiles in only non-competitive events (Saarni 1984), the effect of only sociality on smiles (Schneider and Josephs 1991), or the effect of only culture on smiles (Garrett-Peters and Fox 2007). Moreover, even though our study adopts an existing game paradigm that has been shown to elicit spontaneous facial expression from children, we have contributed new insights into cross-cultural differences in smiling behavior of children. Shahid et al. (2008) only performed a perception study; the claims they made about cultural differences in expression are questionable, as they did not code or analyze the actual facial displays of children. In our study, we have examined the production of smiles displayed by children by means of FACS, substantiating our assertions of cultural differences.

In the present study, children assigned to play the game in pairs were free to pair up with any friend who was physically present at that time; in other words, they were collaborating with an in-group member against the computer. Another research question we have not examined, but is nevertheless interesting, concerns what happens when children compete against other children who are either in-group or out-group members, instead of against a computer. The distinction between in-groups and out-groups is related to the cultural framework of individualism-collectivism, with group membership being more diffused in individualistic societies and more well-defined in collectivistic societies (Triandis et al. 1988). Hence, it is likely that children from collectivistic cultures vary their expression with the group membership of their opponent to a greater extent than children from individualistic cultures. Recently, Van Osch et al. (2016) showed that American medalists were perceived as expressing more pride than Chinese medalists when outperforming in-group competitors (i.e., in American and Chinese national championships, respectively); this cultural difference was not observed for medalists outperforming out-group competitors (i.e., in the Olympics where the defeated were of a nationality other than their own). Researchers with similar interests may consider replicating these findings among children in future studies.

We are aware that some researchers have associated expressions displayed by winners in competitive events to expressions of pride, which involve other nonverbal features such as raised arms or expanded chest (e.g., Aviezer et al. 2012; Tracy and Robins 2004, 2007; Van Osch et al. 2016). Although the game used in our paradigm was competitive in nature, it did not involve the substantial preparations and considerable consequences faced by professional adult athletes in a competition. Therefore, whilst we are confident that our paradigm sufficiently induced feelings and expressions associated with winning and losing, we find it highly unlikely that children participating in our study experienced or displayed pride, a self-conscious emotion whose psychological structure is quite distinct (Tracy and Robins 2004). In the present study, we have focused on the role of smiles in expressive displays of winning and losing, and less on the emotions experienced by children per se. Analyses of other nonverbal features are necessary to determine the specific emotion displayed by children.

To conclude, we set out to investigate the interplay between social context and cultural context on smiles displayed by children upon winning and losing in games. By means of a game paradigm, we have shown that children indeed smile more when they are accompanied by a friend than when they are alone. In line with existing cultural frameworks, we have shown that the effect of sociality on smile intensity is indeed more pronounced among Chinese children than Dutch children. Whereas the intensity of smiles of Chinese pairs is greater than those of Chinese individuals, smile intensity does not differ between Dutch pairs and Dutch individuals.
